# Supplementary figures and images for: N95 respirator hybrid decontamination method using Ultraviolet Germicidal Irradiation (UVGI) coupled with Microwave-Generated Steam (MGS)
Source: PLoS One. 2024 Feb 6;19(2):e0296871. doi: 10.1371/journal.pone.0296871 (PMC10846690; doi:10.1371/journal.pone.0296871)

Decontamination system operation procedure


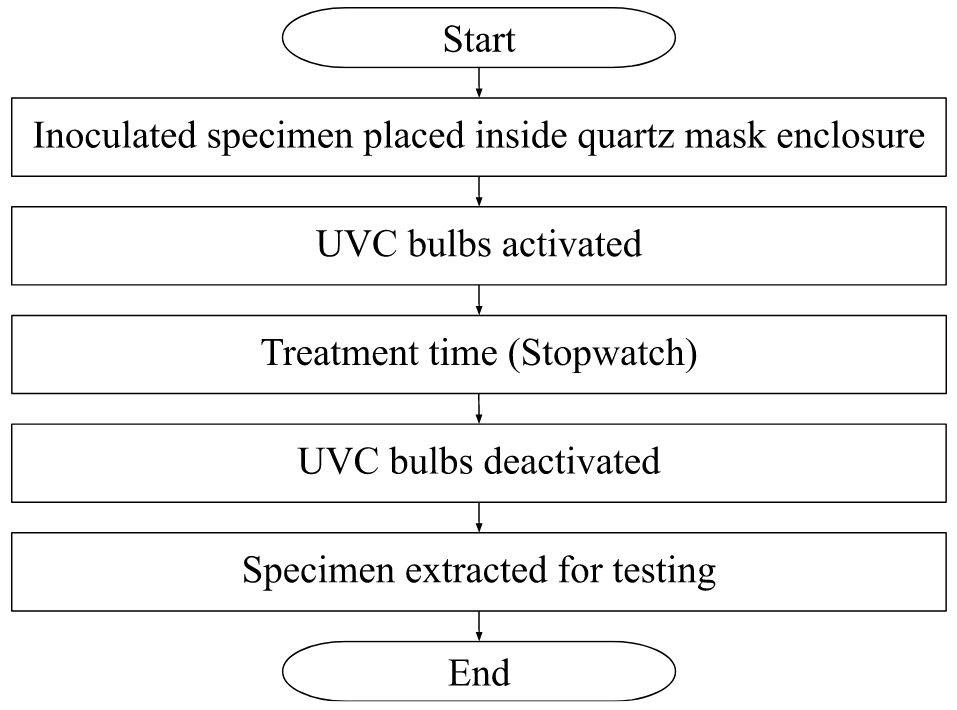
1) UVGI-Only

2) MGS-Only


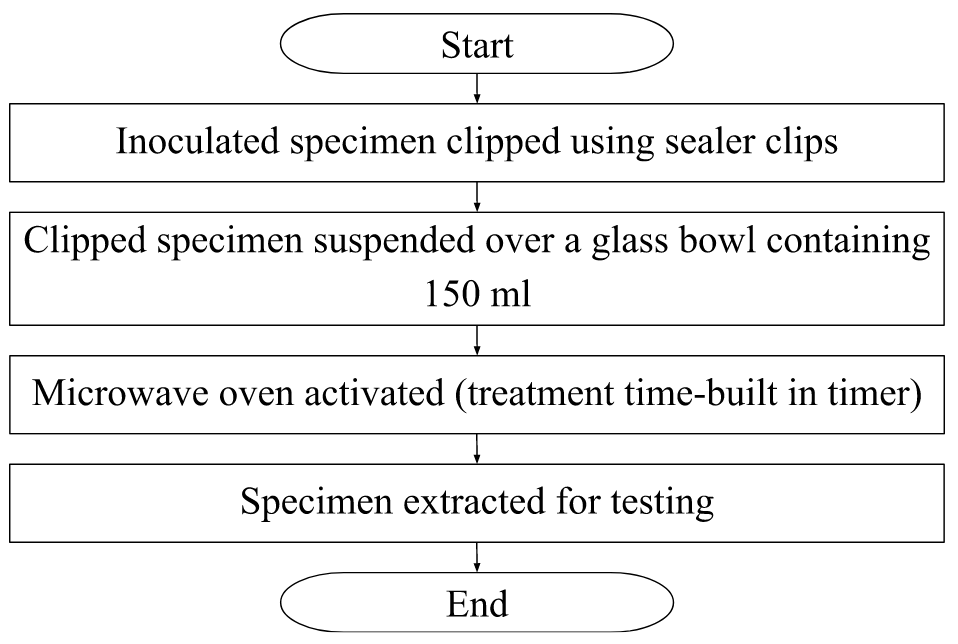


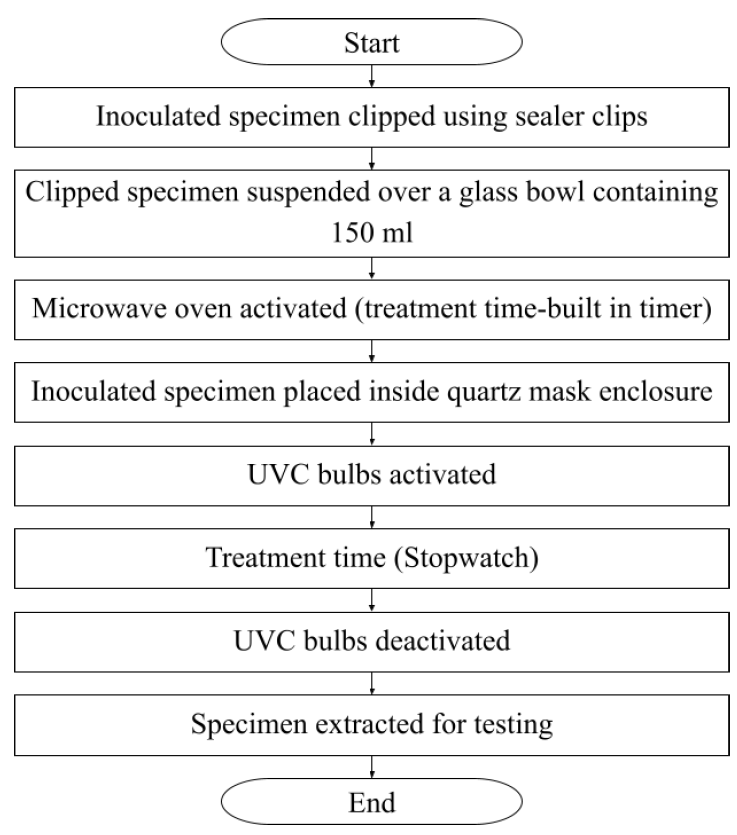
3) Hybrid - UVGI + MGS

MGS

UVGI

Supplement: S1 File — (DOCX) [file pone.0296871.s001.docx]
