# Supplementary material for: N95 respirator hybrid decontamination method using Ultraviolet Germicidal Irradiation (UVGI) coupled with Microwave-Generated Steam (MGS)
Source: PLoS One. 2024 Feb 6;19(2):e0296871. doi: 10.1371/journal.pone.0296871 (PMC10846690; doi:10.1371/journal.pone.0296871)
Supplement: Attachment — Submitted filename: Response to Reviewers.docx [file pone.0296871.s003.docx]

Dear Dr Vittorio Sambri, M.D., Ph.D.,

Academic Editor

PLOS ONE

Thank you for giving us the opportunity to re-submit our manuscript. The manuscript has been revised according to the reviewers’ comments, as follows:

**Reviewer #1:**

**Question 1:** When introducing the term UVGI in the abstract, specify the type of UV eg UVC.

**Answer:** Dear reviewer, thank you for your comment. The **abstract (Pg 2)** section has been modified according to the suggestion.

The Coronavirus Disease 2019 (COVID-19) pandemic has induced a critical supply of personal protective equipment (PPE), especially N95 respirators. Utilizing respirator decontamination procedures to reduce the pathogen load of a contaminated N95 respirator can be a viable solution for reuse purposes. In this study, the efficiency of a novel hybrid respirator decontamination method of ultraviolet germicidal irradiation (UVGI) which utilizes ultraviolet-C (UV-C) rays coupled with microwave-generated steam (MGS) against feline coronavirus (FCoV) was evaluated. The contaminated 3M 1860 respirator pieces were treated with three treatments (UVGI-only, MGS-only, and Hybrid - UVGI + MGS) with variable time. The virucidal activity was evaluated using the TCID50 method. The comparison of decontamination efficiency of the treatments indicated that the hybrid method achieved at least a pathogen log reduction of 4 logs, faster than MGS and UVGI. These data recommend that the proposed hybrid decontamination system is more effective comparatively in achieving pathogen log reduction of 4 logs.

**Question 2:** The use of the word ‘mask’ is confusing. If referring to an N95 respirator, the term ‘respirator’ would be more appropriate and accurate.

**Answer:** Dear reviewer, thank you for your comment. The suggestion is apt and accurate. The **entire revised manuscript** has been modified in line with the term respirator.

**Question 3:** Remember to define acronyms eg. COVID

**Answer:** Dear reviewer, thank you for your comment. The first instance of the COVID term has been defined in the **abstract (Pg 2)** and **introduction (Pg 2)** section.

**Abstract (Pg 2)**

The Coronavirus Disease 2019 (COVID-19) pandemic has induced a critical supply of personal protective equipment (PPE), especially N95 respirators. Utilizing respirator decontamination procedures to reduce the pathogen load of a contaminated N95 respirator can be a viable solution for reuse purposes. In this study, the efficiency of a novel hybrid respirator decontamination method of ultraviolet germicidal irradiation (UVGI) which utilizes ultraviolet-C (UV-C) rays coupled with microwave-generated steam (MGS) against feline coronavirus (FCoV) was evaluated. The contaminated 3M 1860 respirator pieces were treated with three treatments (UVGI-only, MGS-only, and Hybrid - UVGI + MGS) with variable time. The virucidal activity was evaluated using the TCID50 method. The comparison of decontamination efficiency of the treatments indicated that the hybrid method achieved at least a pathogen log reduction of 4 logs, faster than MGS and UVGI. These data recommend that the proposed hybrid decontamination system is more effective comparatively in achieving pathogen log reduction of 4 logs.

**Introduction (Pg 2)**

The unprecedented Coronavirus Disease 2019 (COVID-19) pandemic has alarmed the world regarding the need for respiratory protection against airborne diseases. This urgent necessity for respiratory protection resulted in a rapid increase in demand for personal protective equipment (PPE), particularly face masks. Healthcare professionals who often handle COVID-related illnesses rely solely on PPE, particularly N95 respirators, to safeguard against this virus (1). The N95-type respirator is applicable and widely used in healthcare applications (2).

**Question 4:** On Pg.4 – the term pathogens are denatured is used. Feels. Proteins are denatured and pathogens are typically described as inactivated, killed, lysed, etc.

**Answer:** Dear reviewer, thank you for your comment. The suggestion is apt and accurate. The introduction (Pg 4) section has been modified according to the suggestion.

**Introduction (Pg 4)**

MGS is among the decontamination techniques investigated currently that show great potential. MGS procedure is a type of steam treatment that uses microwave oven sterilization coupled with the presence of moisture. Water is capable of absorbing microwave energy, lowering the risk of harming N95 respirator components (18). Notably, MGS treatment is well known for quick disinfection and affordability where microwave oven has been a common household item nowadays. In this treatment, pathogens are inactivated by generating heat within water molecules using microwave energy. Initial studies demonstrate that MGS was capable of producing outstanding microbiological outcomes with the preservation of the integrity of the treated respirators at a relatively shorter treatment time (19-22). Fisher et al. reported an excellent 3-log reduction in MS2 using a 90-second microwave treatment. This study described an MGS procedure using steam bags and a commercially available microwave oven. Notably, filtration performance was preserved post-decontamination (19).

**Question 5:** Interesting mention of the drawbacks on page 4. I would recommend moving these to the discussion.

**Answer:** Dear reviewer, thank you for your comment.The details on the drawbacks of the decontamination systems have been moved to the discussion (Pg 10-11) section.

**Discussion (Pg 10-11)**

To our knowledge, this current study is the first to report an evaluation of the efficiency of the hybrid decontamination (UVGI + MGS) on an N95 respirator against FCoV. The key idea of approaching this hybrid decontamination technique in this study was to find a treatment that offers rapid and safe decontamination. As the results suggest, hybrid decontamination using UVGI and MGS can significantly save time and cost in decontaminating respirators up to a safe level. Both UVGI and MGS exhibit great potential as efficient decontamination systems where a higher log reduction value is offered at a relatively shorter treatment time. In comparison with the other available treatments, these two system does not leave any residue which could pose a safety threat to the user. However, several disadvantages of these systems have to be handled with caution. Firstly, as the previous studies suggest, each decontamination procedure has its drawback of affecting the integrity and performance of the respirator when exposed to longer treatment times (14, 20, 22, 27). Therefore, multiple decontamination cycles must be monitored closely. The hybrid treatment design could potentially alleviate the dependence on a single system for complete sterilization which could assist in preserving the functionality of the respirator for reuse. Secondly, a basic level of expertise is required in handling the UVGI device. In addition, the limited space of the microwave oven constricts the number of respirators that can be decontaminated in one cycle (1).

**Question 6:** Viral Kill Time Assay methods – please describe how the masks were ‘infected’. Eg 100 ul was pipetted over the entire surface of the mask piece?

**Answer:** Dear reviewer, thank you for your comment. The **materials and methods** **(Pg 6)** section has been modified according to the suggestion.

**Materials and methods (Pg 6)**

***Viral Kill Time Assay***

To perform the viral inactivation assay, each respirator piece (4x2 cm) was covered with 100 µl of FCoV suspension with a titre of 1x106 TCID50/ML according to the test standard ISO18184-2019 (23). Subsequently, the infected respirators were subjected to three different treatments for disinfection. These treatments included exposure to UVGI, MGS, or a hybrid procedure using a combination of UVGI and MGS at different time points. A control group, which was not exposed to any treatments, was included for comparison purposes.

To recover the virus from the infected respirators, rinsing was performed using 1 ml of DMEM containing 2% FBS. The recovered virus was then transferred to a microcentrifuge tube, and a 10x serial dilution was performed. The different dilutions of the virus were used to infect the CRFK cells seeded in a 96-well plate. The cells were incubated for 72 hours until cytopathic effects (CPE) developed. Fixation and staining of the infected cells were carried out using a mixture of paraformaldehyde and crystal violet. The virus titer was determined using the Spearman-Karber method and expressed as a tissue culture infectious dose 50% (TCID50/ml). The virucidal activity was assessed by calculating the difference in logarithmic titer (Δ log10 TCID50/ml) between the virus control and the test virus.

**Question 7:** There needs to be a description in the methods of how the dual exposure to MGS and UVGI happened. Was it sequential? Which treatment occurred first?

**Answer:** Dear reviewer, thank you for your comment. Since this comment is similar to Question 14 from Reviewer 2, the answer is consolidated together and discussed more in detail. Kindly please review the comment in the said section.

**Question 8:** How is the hybrid method is a significant time or cost savings as purported by the authors? Hybrid method: 3 logs was 36 seconds, 4 logs 39 seconds. MGS: 4 logs was 45 seconds; UVGI 3 logs after 9 seconds and 4 logs after 100 seconds. Without a thorough explanation of the hybrid method procedure, it is hard to imagine the total operating time would be less using the hybrid method, including operator time to operate two different decon machines. Please provide an explanation.

**Answer:** Dear reviewer, thank you for your comment. A detailed flow chart of the operational procedure has been added in the supporting information section denoted as **S1 File. Decontamination system operation procedure.** The authors would like to clarify that this study is a fundamental study which scope is to find the least decontamination time possible using the hybrid method in comparison with other single treatments. In that case, the hybrid method offers faster treatment time in comparison. The reported 6-second advantage (hybrid compared to MGS 4-log) could be significantly higher in the real application of multiple cycles or a higher number of respirators decontaminated in comparison with other methods. In addition, significantly shorter treatment times could result in lower overall operation costs. Nevertheless, this real application was not included in the scope of the current research.

**Reviewer #2**:

**Question 1:** The title of the manuscript is suitable and clear. (Title)

**Answer:** Dear reviewer, thank you for your comment.

**Question 2:** Abstract is an adequate summary of the work presented. (Abstract)

**Answer:** Dear reviewer, thank you for your comment.

**Question 3:** The word sterilization should be changed in disinfection when you use UVC (Introduction)

**Answer:** Dear reviewer, thank you for your comment. The suggestion is apt and accurate. The introduction (Pg 3-4) section has been modified according to the suggestion.

**Introduction (Pg 3-4)**

Since the late 19th century, UVGI treatment has been used and practiced in disinfection applications all over the world (5). UVGI procedure utilizes a UVC-producing source such as bulbs to kill viral, bacterial, and fungal organisms (6). By causing damage to their DNA, UV-C radiation either kills or inactivates microorganisms (5). The degradation of DNA structure by UV-C can be due to the formation of photoproducts, free radicals generation, and strand breaks which include either single or double DNA strand breaks (7, 8). Related investigations demonstrate the UVGI decontamination system's effectiveness against coronaviruses like SARS-CoV-2 and MERS-CoV (9, 10). Ozog et al. produced excellent virus inactivation results of at least 3 log reductions in viable SARS-CoV-2 using a dosage of 1.5 J/cm2 (11). Furthermore, a log reduction of at least 3 was achieved by Geldert et al. where the specimen was treated by 0.05 -0.5 J/cm² of UVC dosage (12). High virucidal activity while maintaining the integrity of the treated specimen is one of the key advantages of the UVGI treatment. The degradation of N95 respirators associated with UVGI treatment can be explained by the administration of excessive UV dosage to a specimen. Several reports exhibit excellent log reduction results produced while preserving the integrity and performance of a treated mask (11, 13-17). In addition, an excessive UVC dosage of 950 J/cm² on the 3M 1860 N95 respirator was tested by Lindsley et al. This study concludes that at the specified high dosage the filtration performance was minimally affected and flow resistance was not affected (14).

**Question 4:** “3M indicates that reducing”: I think it is useful to write 3M company (Introduction)

**Answer:** Dear reviewer, thank you for your comment. The suggestion is apt and accurate. The correct term has been modified.

**Introduction (Pg 3)**

Consequently, a respirator decontamination technique for reuse could assist in resolving the rising demand for respirator supply. In order to increase the reusability of respirators, a safe respirator decontamination technique may help reduce the pathogen burden of a contaminated respirator. The 3M company indicates that reducing the pathogen burden of the respirator through a decontamination procedure is one of the key criteria for reusing a respirator (3). According to the National Institute of Occupational Safety and Health (NIOSH), UVGI, hydrogen peroxide vapor (HPV), and moist heat are recommended to be efficient decontamination treatments against pathogens (4).

**Question 5:** “HPV” : an acronym should be written out in full the first time it is used. (Introduction)

**Answer:** Dear reviewer, thank you for your comment. The first instance of the HPV term has been defined in the introduction (Pg 3) section.

**Introduction (Pg 3)**

Consequently, a respirator decontamination technique for reuse could assist in resolving the rising demand for respirator supply. In order to increase the reusability of respirators, a safe respirator decontamination technique may help reduce the pathogen burden of a contaminated respirator. The 3M company indicates that reducing the pathogen burden of the respirator through a decontamination procedure is one of the key criteria for reusing a respirator (3). According to the National Institute of Occupational Safety and Health (NIOSH), UVGI, hydrogen peroxide vapor (HPV), and moist heat are recommended to be efficient decontamination treatments against pathogens (4).

**Question 6:** “By causing damage to their DNA, UV-C radiation either kills or inactivates microorganisms (5).” : On this subject, I believe there are more effective citations, for example Kciuk M, Marciniak B, Mojzych M, Kontek R. Focus on UV-Induced DNA Damage and Repair-Disease Relevance and Protective Strategies. Int J Mol Sci. 2020 Oct 1;21(19):7264. doi: 10.3390/ijms21197264. PMID: 33019598; PMCID: PMC7582305 or Rastogi RP, Richa, Kumar A, Tyagi MB, Sinha RP. Molecular mechanisms of ultraviolet radiation-induced DNA damage and repair. J Nucleic Acids. 2010 Dec 16;2010:592980. doi: 10.4061/2010/592980. PMID: 21209706; PMCID: PMC3010660. (Introduction)

**Answer:** Dear reviewer, thank you for your comment. Excellent suggestion of articles. The new articles have been added to the introduction (Pg 3-4) section.

**Introduction (Pg 3-4)**

Since the late 19th century, UVGI treatment has been used and practiced in disinfection applications all over the world (5). UVGI procedure utilizes a UVC-producing source such as bulbs to kill viral, bacterial, and fungal organisms (6). By causing damage to their DNA, UV-C radiation either kills or inactivates microorganisms (5). The degradation of DNA structure by UV-C can be due to the formation of photoproducts, free radicals generation, and strand breaks which include either single or double DNA strand breaks (7, 8). Related investigations demonstrate the UVGI decontamination system's effectiveness against coronaviruses like SARS-CoV-2 and MERS-CoV (9, 10). Ozog et al. produced excellent virus inactivation results of at least 3 log reductions in viable SARS-CoV-2 using a dosage of 1.5 J/cm2 (11). Furthermore, a log reduction of at least 3 was achieved by Geldert et al. where the specimen was treated by 0.05 -0.5 J/cm² of UVC dosage (12). High virucidal activity while maintaining the integrity of the treated specimen is one of the key advantages of the UVGI treatment. The degradation of N95 respirators associated with UVGI treatment can be explained by the administration of excessive UV dosage to a specimen. Several reports exhibit excellent log reduction results produced while preserving the integrity and performance of a treated mask (11, 13-17). In addition, an excessive UVC dosage of 950 J/cm² on the 3M 1860 N95 respirator was tested by Lindsley et al. This study concludes that at the specified high dosage the filtration performance was minimally affected and flow resistance was not affected (14).

**Question 7:** “In addition, the limited space of the microwave oven constricts the number of masks that can be decontaminated in one cycle (1).” : Is the number in brackets a citation or is it the number of masks that can be decontaminated in one cycle? (Introduction)

**Answer:** Dear reviewer, thank you for your comment. The number in the bracket refers to a citation (Gopalan T, Mohd Yatim RaA, Muhamad MR, Mohamed Nazari NS, Awanis Hashim N, John J, et al. Decontamination Methods of N95 Respirators Contaminated with SARS-CoV-2. Sustainability. 2021;13(22):12474). This statement has been moved to the **discussion (Pg 10-11)** section as per the suggestion by Reviewer 1.

**Question 8:** The timing of the study (start, run and end) should be described. (Method)

**Answer:** Dear reviewer, thank you for your comment. Since this comment is similar to Question 14 from Reviewer 2, the answer is consolidated together and discussed more in detail. Kindly please review the comment in the said section.

**Question 9:** The design/type of the study is missing (Method)

**Answer:** Dear reviewer, thank you for your comment. The design of the study has been added to the materials and methods (Pg 5) section.

**Materials and methods (Pg 5)**

***Design of the Study***

This specific study is an experimental research. An extensive literature review was conducted on various respirator decontamination techniques which include UVGI, MGS, HPV, dry heat, moist heat, and ethanol (1). The selection of UVGI and MGS was based on the ease of incorporating both treatments in one cycle and excellent decontamination efficiency against pathogens. The parameter design was developed using estimation from existing reported data in the reviewed articles.

**Question 10:** “5 UVC bulbs (TUV PL-S 9W; Phillips)” : I think it is useful to specify the wavelength. The manufacturer states a peak of 253.7 nm in the data sheet, does the value in the prototype correspond to this? (Method)

**Answer:** Dear reviewer, thank you for your comment. A measurement process of the wavelength of the UV rays was not performed. This is due to the bulbs utilized in this prototype were newly acquired. Therefore, all the specifications and calculations were based on the manufacturer’s data sheet which indicates a peak of 253.7 nm. The additional details regarding the wavelength of the bulbs have been modified in the materials and methods (Pg 7) section.

**Materials and methods (Pg 7)**

UVGI Decontamination System

A custom mask sterilizer machine equipped with a UVGI decontamination system measuring 270 x 315 x 470 mm (LxHxW) was designed and fabricated for this study (Fig 1-A). The machine was made up of Stainless Steel 304 for the majority of the fabricated parts where a few small parts were fabricated using Aluminium 5083. A special mask enclosure was made using acryl and quartz glass. The selection of quartz glass was based on its ability to allow UVC rays to penetrate through and reach the contaminated specimen. The design of the UVGI system in the machine was constructed using 5 UVC bulbs (TUV PL-S 9W; Phillips), capable of producing rays with a peak at 253.7 nm. These bulbs were arranged in a manner where 3 focus on the exterior meanwhile 2 focus on the interior of the respirator specimen (Fig 1-B). Only 3 upper bulbs were utilized for this study which generates a combined irradiance value of at least 7.5 mW/cm2 at 10 cm. Polished reflective stainless steel sheets were placed around the bulbs to maximize the reflection of ultraviolet rays. Equation 1 was used to calculate the required UV dosage for each designed parameter of the tests (24).

**Question 11:** “This 1200W-rated microwave oven is capable of producing an output power of 600 - 650 W.” : It is useful to specify the power used for the experiment, as the product data sheet states a maximum output power of 800 Watts with 6 different levels. (Method)

**Answer:** Thank you for the comment. The output power of 600 - 650 W as stated in the manuscript was calculated using the determination of microwave power output method according to IEC 60705. The formula and data have been attached in the supporting information section denoted as S2 File. Determination of microwave power output.

**Question 12:** There is no description of how the three tests are carried out, particularly in relation to the combined test. Which decontamination method is used first in the combined test? Was there any downtime between methods? In my opinion, a step-by-step description of the procedure for the three tests is necessary. (Method)

**Answer:** Dear reviewer, thank you for your comment. Since this comment is similar to Question 14 from Reviewer 2, the answer is consolidated together and discussed more in detail. Kindly please review the comment in the said section.

**Question 13:** Repetitions of the tests are not present (Method)

**Answer:** Dear reviewer, thank you for your comment. Since this comment is similar to Question 16 from Reviewer 2, the answer is consolidated together and discussed more in detail. Kindly please review the comment in the said section.

**Question 14:** Provide a more detailed description of the decontamination techniques used. Adding explanatory figures about the techniques employed and the execution protocols could make the reading more understandable for the readers. (Method)

**Answer:** Dear reviewer, thank you for your comment. A detailed flow chart of the operational procedure has been added in the supporting information section denoted as **S1 File. Decontamination system operation procedure.**

**Materials and methods (Pg 6-7)**

***Decontamination Treatments***

A total of 3 types of decontamination treatments were investigated in this research which include UVGI-only, MGS-only, and Hybrid (UVGI + MGS). The detailed operational procedures of these treatments are available in the S1 File.

**Question 15:** Include the brand names of the culture media and solutions used, along with the legal headquarters of the manufacturing company. (Method)

**Answer:** Dear reviewer, thank you for your comment. The materials and methods (Pg 5-6) section has been modified according to the suggestion.

**Materials and methods (Pg 5-6)**

***Cells and Virus***

In this study, feline coronavirus (FCoV) obtained from the Tropical Infectious Diseases Research & Education Centre was used. The FCoV was propagated in the CRFK cell line using Dulbecco's Modified Eagle Medium (DMEM) (HyClone, Thermo Scientific, USA) supplemented with 2% Fetal Bovine Serum (FBS) (Gibco, Thermo Scientific, USA). Following the observation of cytopathic effects in the cells, the virus was harvested and stored at -80°C for future use. The viral titers were determined using the TCID50 method.

**Question 16:** Table 1 is not consistent with the log reduction. The results are inaccurate. How many times were repeated the tests? (Results)

**Answer:** Dear reviewer, thank you for your comment. We would like to confirm that each sample was tested 3 times and we have a few repeated data for one specific log reduction data. We did not repeat all the tests as it is expensive. However, data from hybrid test results confirmed repeatability.

**Results (Pg 8)**

The maximum detectable log reduction for this test was determined to be a 4 log reduction and each sample was tested three times to produce one log reduction data for the specific sample. The UVGI-only tests resulted in log reduction exceeding the detection limit of 4 logs after being treated with a UVC dosage of 0.75 J/cm^2^ (Table 1). This UVC dosage is equivalent to 100 seconds of UV exposure. In addition, 3 log reduction was observed at a dosage of 0.0675 J/cm^2^. 2 data were omitted due to ambiguity.

**Question 17:** “FFR” : An acronym should be written out in full the first time it is used. (Discussion)

**Answer:** Dear reviewer, thank you for your comment. The first instance of the FFR term has been defined in the discussion (Pg 9-10) section.

**Discussion (Pg 9-10)**

In this study, the efficiency of a hybrid respirator decontamination technique of UVC coupled with MGS against FCoV was evaluated in comparison to single treatments of UVGI and MGS. Results from the tests indicate excellent virucidal activity incorporated with the proposed hybrid decontamination technique. The hybrid decontamination technique achieved pathogen reduction of at least 4 logs faster than the single treatments of UVGI and MGS (Fig 3). The reported ≥4 log reduction in this current study is considered significant as 3 logs are the minimum required pathogen reduction to completely disinfect a contaminated filtering facepiece respirator (FFR) (24, 25).

**Question 18:** it should be verified what would happen, using microwaves, to the masks on a mechanical level. I believe that heating could have a negative impact. It is plausible that there could be an alteration in the fiber structure. This aspect should be investigated. (Discussion)

**Answer:** Dear reviewer, thank you for your comment. The discussion (Pg 11-12) section has been modified according to the suggestion.

**Discussion (Pg 11-12)**

The considerations for the future scope of this current study need to be noted. Firstly, the current study does not report on performance and integrity degradation incorporated with respirator decontamination. Referring to UVGI decontamination on 3M 1860 respirator, Lindsley et al.reported unaffected flow resistance at UV dosages up to 950 J/cm2 (14), and Lore et al. reported no signification changes to filter performance at a dosage of 1.8 J/cm2 (20). Referring to MGS decontamination on 3M 1860 respirator, Fisher et al. reported preserved filtration efficiency after 1 decontamination cycle of 90 seconds (19), and Zulauf et al. reported preservation of fit, seal, and filtration performance for up to 20 cycles of 180 seconds (22). In comparison, our current study utilizes significantly lower UV dosages and MGS treatment time which could eliminate the possibility of significant integrity or performance impact on the respirator. Secondly, the irradiance value of a UVC source which indicates the energy per unit area directly proportional to the UV dosage value. Sufficient dosage value is crucial for complete decontamination. Future investigations could utilize UVC setup with higher irradiance values which could offer relatively shorter treatment time to achieve specific log reduction values. Thirdly, the resistance of the tested organism plays a major role in the dosage of treatment required for complete decontamination. FCoV used in this study which is an enveloped virus is one of the least resistant organisms against disinfectants. More resilient microorganisms such as non-enveloped viruses and bacterial spores may require higher treatment time for complete decontamination (28).

**Question 19:** The analyzed sample is relatively small. Is it conceivable that the distribution of microwaves inside the chamber is uniform? If not, there could be a different level of disinfection on the mask. (Discussion)

**Answer:** Dear reviewer, thank you for your comment. The materials and methods (Pg 7-8) section has been modified according to the suggestion.

**Materials and methods (Pg 7-8)**

*MGS Decontamination System*

A commercially available microwave oven (MG23K3513GK; Samsung) was selected for MGS-based decontamination tests. This 1200W-rated microwave oven is capable of producing an output power of 600 - 650 W (S2 File). This microwave is equipped with a turntable which ensures uniform heating and steam generation (22). The decontamination setup (Fig 2) for this technique was inspired by the investigation by Zulauf et al (22). The inoculated respirator piece was fixed in between 2 parallel sealer clips. This fixture was suspended over a bowl of 150 ml water which was subjected to a specified microwave treatment time.

**Question 20:** Another aspect that should be investigated is the energy distribution of UVC light inside the chamber when a mask is inserted. Would the energies at play per unit of surface area change? These aspects should be commented on. (Discussion)

**Answer:** Dear reviewer, thank you for your comment. The discussion (Pg 11-12) section has been modified according to the suggestion.

**Discussion (Pg 11-12)**

The considerations for the future scope of this current study need to be noted. Firstly, the current study does not report on performance and integrity degradation incorporated with respirator decontamination. Referring to UVGI decontamination on 3M 1860 respirator, Lindsley et al.reported unaffected flow resistance at UV dosages up to 950 J/cm2 (14), and Lore et al. reported no signification changes to filter performance at a dosage of 1.8 J/cm2 (20). Referring to MGS decontamination on 3M 1860 respirator, Fisher et al. reported preserved filtration efficiency after 1 decontamination cycle of 90 seconds (19), and Zulauf et al. reported preservation of fit, seal, and filtration performance for up to 20 cycles of 180 seconds (22). In comparison, our current study utilizes significantly lower UV dosages and MGS treatment time which could eliminate the possibility of significant integrity or performance impact on the respirator. Secondly, the irradiance value of a UVC source which indicates the energy per unit area directly proportional to the UV dosage value. Sufficient dosage value is crucial for complete decontamination. Future investigations could utilize UVC setup with higher irradiance values which could offer relatively shorter treatment time to achieve specific log reduction values. Thirdly, the resistance of the tested organism plays a major role in the dosage of treatment required for complete decontamination. FCoV used in this study which is an enveloped virus is one of the least resistant organisms against disinfectants. More resilient microorganisms such as non-enveloped viruses and bacterial spores may require higher treatment time for complete decontamination (28).

**Question 21:** The research is solely based on testing one virus. The resistance to other pathogens could be at least estimated based on the literature and the presented results. The hypothesis of the technique used is that it may be effective with other microbial species as well. (Discussion)

**Answer:** Dear reviewer, thank you for your comment. The discussion (Pg 11-12) section has been modified according to the suggestion.

**Discussion (Pg 11-12)**

The considerations for the future scope of this current study need to be noted. Firstly, the current study does not report on performance and integrity degradation incorporated with respirator decontamination. Referring to UVGI decontamination on 3M 1860 respirator, Lindsley et al.reported unaffected flow resistance at UV dosages up to 950 J/cm2 (14), and Lore et al. reported no signification changes to filter performance at a dosage of 1.8 J/cm2 (20). Referring to MGS decontamination on 3M 1860 respirator, Fisher et al. reported preserved filtration efficiency after 1 decontamination cycle of 90 seconds (19), and Zulauf et al. reported preservation of fit, seal, and filtration performance for up to 20 cycles of 180 seconds (22). In comparison, our current study utilizes significantly lower UV dosages and MGS treatment time which could eliminate the possibility of significant integrity or performance impact on the respirator. Secondly, the irradiance value of a UVC source which indicates the energy per unit area directly proportional to the UV dosage value. Sufficient dosage value is crucial for complete decontamination. Future investigations could utilize UVC setup with higher irradiance values which could offer relatively shorter treatment time to achieve specific log reduction values. Thirdly, the resistance of the tested organism plays a major role in the dosage of treatment required for complete decontamination. FCoV used in this study which is an enveloped virus is one of the least resistant organisms against disinfectants. More resilient microorganisms such as non-enveloped viruses and bacterial spores may require higher treatment time for complete decontamination (28).

**Question 22:** With regard to the limitations of the work, more data would have given the study greater significance and, in addition, repeating the tests on several samples for each exposure time would have avoided the ambiguity of the data found in the UVGI-only decontamination test. (Discussion)

**Answer:** Dear reviewer, thank you for your comment. Since this comment is similar to Question 16 from Reviewer 2, the answer is consolidated together and discussed more in detail. Kindly please review the comment in the said section.

**Question 23:** It should be noted: UVGI reaches 3 logs (minimum required pathogen reduction to completely disinfect a contaminated) in about a quarter of the time compared to UVGI + MGS. (Conclusion)

**Answer:** **Dear reviewer, thank you for your comment. The conclusion (Pg 12) section has been modified according to the suggestion.**

**Conclusion (Pg 12)**

Effective FFR decontamination procedures are crucial in critical pandemic scenarios such as COVID-19. UVGI and MGS exhibit great potential in treating contaminated N95 respirators. Our study concludes that the hybrid decontamination procedure of UVGI coupled with MGS is more effective than single treatments of UVGI and MGS in achieving a 4-log pathogen load reduction. In addition, UVGI was able to produce a 3-log reduction in a quarter of the time managed by the hybrid decontamination procedure. Implementation of this procedure will require further investigations on models, filtration performance, and fit testing of the treated respirators.

**Question 24:** Cited References are relevant and sufficient to place the work in context. (Have a look at the suggested work in the Introduction section.) (References)

**Answer:** Dear reviewer, thank you for your comment. The new journal references are added accordingly.

**Question 25:** The tables and graph are clear. Graph needs better quality (Tables and Figures)

**Answer:** Dear reviewer, thank you for your comment. A new graph has been produced using the PACE tool.

**Question 26:** For the sake of clarity, the legend should be reproduced below the relevant figure. (Tables and Figures)

**Answer:** Dear reviewer, thank you for your comment. Figure 1 and 2 has been reproduced using legends attached to the figure as per suggestion. Figures have been produced using the PACE tool.
